# A Novel Anti-Cadherin-17 Monoclonal Antibody, Ca_17_Mab-5, for Multiple Applications

**DOI:** 10.3390/antib15040059

**Published:** 2026-07-10

**Authors:** Reina Ito, Hiroyuki Suzuki, Kenichiro Ishikawa, Kazutake Yagi, Akira Ohkoshi, Yukio Katori, Mika K. Kaneko, Yukinari Kato

**Affiliations:** 1 Department of Antibody Drug Development, Tohoku University Graduate School of Medicine, 2-1 Seiryo-machi, Aoba-ku, Sendai 980-8575, Japan; ito.reina.r7@dc.tohoku.ac.jp (R.I.); mika.kaneko.d4@tohoku.ac.jp (M.K.K.); 2 Department of Otolaryngology, Head and Neck Surgery, Tohoku University Graduate School of Medicine, 1-1 Seiryo-machi, Aoba-ku, Sendai 980-8575, Japan; ken.ishikawa.r3@dc.tohoku.ac.jp (K.I.); kazutake.yagi.e4@tohoku.ac.jp (K.Y.); ohkoshia@hotmail.com (A.O.); yukio.katori.d1@tohoku.ac.jp (Y.K.)

**Keywords:** Cadherin-17, CDH17, monoclonal antibody, Cell-Based Immunization and Screening, flow cytometry, immunohistochemistry

## Abstract

Background/Objectives: Cadherin-17 (CDH17, LI-cadherin) is a non-classical cadherin with an atypical structure and unique functions. CDH17 expression is restricted to normal intestinal epithelium. Furthermore, CDH17 functions as an oncoprotein that promotes tumor migration and invasion in colorectal, gastric, and pancreatic cancers. Therefore, CDH17 is an important diagnostic marker and therapeutic target. The CDH17-directed strategies, including monoclonal antibodies (mAbs), bispecific Abs, antibody–drug conjugates (ADCs), and chimeric antigen receptor (CAR) T cells, have been evaluated in preclinical and clinical studies. Therefore, developing mAbs that specifically recognize cell surface-expressing CDH17 is essential for advancing both tumor diagnosis and therapy. Methods: Anti-human CDH17 mAbs (named Ca_17_Mabs) were developed by immunizing a mouse with CDH17-overexpressed cells and a high-throughput screening using flow cytometry. Results: Among Ca_17_Mabs, a clone, Ca_17_Mab-5 (IgG_1_, κ) specifically recognized CDH17-overexpressed Chinese hamster ovary-K1 (CHO/CDH17) cells with no detectable cross-reactivity to 21 other CDHs by flow cytometry. Ca_17_Mab-5 also detected endogenous CDH17 in human colorectal cancer cell lines, COLO201 and COLO205. The apparent dissociation constant (*K*_D_) values of Ca_17_Mab-5 for CHO/CDH17 and COLO205 were estimated as 1.5 × 10^−8^ M and 1.3 × 10^−8^ M, respectively. Furthermore, Ca_17_Mab-5 detected endogenous CDH17 by Western blotting. In immunohistochemistry, Ca_17_Mab-5 exhibited clear membranous staining in normal colon epithelium, colorectal, gastric, and pancreatic cancers. Conclusions: Ca_17_Mab-5 is a versatile tool for detecting CDH17 and has potential for tumor diagnosis.

## 1. Introduction

Cadherins (CDHs) play crucial roles in cell–cell adhesion and the maintenance of tissue architecture [1]. Classical cadherins have five extracellular cadherin (EC) repeats, a single transmembrane domain, and a cytoplasmic domain with highly conserved binding regions for the armadillo proteins, including β-catenin and p120-catenin [1]. The EC repeats, originally identified in the extracellular region of the classical type I cadherin CDH1/E-cadherin [2], bind to Ca^2+^ and mediate homophilic interactions that are important for cell sorting into different compartments during organogenesis [3]. In epithelia, CDH1 forms adherens junctions via homophilic interactions, which define the features of epithelial sheets and apical adhesive structures [4]. The ability to interact with cytoplasmic armadillo proteins is a key function of classical CDHs. p120 catenin directly associates with the membrane-proximal region of the CDH cytoplasmic tail, and β-catenin serves as a scaffold to anchor α-catenin [5]. The cadherin–catenin complex associates directly or indirectly with signaling molecules, scaffolding proteins, and cytoskeletal regulators such as filamentous actin [5].

Cadherin-17 (CDH17), also known as liver-intestine CDH (LI-CDH) or seven-domain (7D) CDH, is a non-classical CDH with seven EC repeats, a single transmembrane domain, and a short cytoplasmic domain that lacks armadillo protein-binding motifs [6,7,8]. The tissue distribution of CDH17 varies across species. CDH17 is expressed in the liver and intestine in rats. In mice and humans, expression is restricted to small intestinal and colonic epithelial cells, with no detectable expression in the liver or stomach [9].

CDH17 plays a crucial role in intestinal barrier function [6]. CDH17-knockout (KO) mice displayed enhanced epithelial permeability in the small intestine and colon in vitro and in vivo models. Moreover, CDH17-KO mice exhibited an increased susceptibility to colitis induced by dextran sulfate sodium (DSS) [10]. In the DSS and azoxymethane-induced colorectal cancer model, CDH17-KO mice showed increased tumor formation and progression in the intestine [10]. Therefore, CDH17 is essential for intestinal homeostasis by limiting intestinal epithelial permeability and acts as a tumor suppressor in colitis-associated tumorigenesis.

Meanwhile, CDH17 is upregulated in colorectal cancer [11] and has been identified as a key mediator of metastatic progression and poor patient survival [12] through interactions with unique binding partners [13,14]. CDH17 binds to the desmosomal cadherin desmocollin-1 (DSC1) and indirectly interacts with actin filaments through the DSC1/p120–catenin complex, thereby promoting migration and invasiveness in CDH17-positive colorectal cancer [15]. CDH17 also interacts with α2β1 integrin via the RGD motif in its ectodomain, thereby promoting colorectal cancer liver metastasis [12,16]. Furthermore, a global transcriptome analysis revealed that silencing CDH17 in metastatic colorectal cancer cell lines markedly reduced the intestinal cancer stem cell marker LGR5, which led to attenuation of Wnt/β-catenin signaling, suppression of stemness-related genes, and consequent impairment of stem cell phenotypes [17].

CDH17 has been considered a promising therapeutic target for preventing metastatic progression in colorectal cancer, with approaches, including monoclonal antibodies (mAbs), bispecific Abs, antibody–drug conjugates (ADCs), and chimeric antigen receptor (CAR)-T [18,19]. CDH17-targeting ADCs have been developed and evaluated in clinical studies. TORL-3-600 is a humanized ADC with an MMAE payload that entered a phase I clinical trial for the treatment of advanced colorectal cancer (NCT05948826) [20]. AMT-676 [21] and YL217 [22] are humanized ADCs with topoisomerase I inhibitor payloads that have entered phase I clinical trials for the treatment of advanced solid tumors (NCT06400485 and NCT06859762, respectively). Furthermore, a T-cell engager cabotamig (CD3 × CDH17 bispecific Ab, NCT05411133) and anti-CDH17 CAR-T therapies (NCT06055439, NCT06501183, and NCT07216560) have been evaluated in clinical studies.

MAbs that detect CDH17 have been developed for Western blotting or immunohistochemistry (IHC). However, suitable anti-CDH17 mAbs for flow cytometry and IHC have been limited. We have developed the Cell-Based Immunization and Screening (CBIS), which includes immunizing antigen-overexpressed cells and high-throughput flow cytometry-based screening to generate highly versatile and specific mAbs. Using the CBIS method, we have developed anti-CDH1 [23], anti-CDH5/VE-cadherin [24], anti-CDH13 [25], and anti-CDH15/M-cadherin [26] mAbs for flow cytometry, Western blotting, and IHC. MAbs obtained from the CBIS method typically recognize conformational epitopes and are applicable in flow cytometry. Importantly, some of them are also applicable in Western blotting and IHC. In this study, we developed highly versatile anti-CDH17 mAbs by the CBIS method.

## 2. Materials and Methods

### 2.1. Cell Lines

Human glioblastoma (GBM) LN229, human colorectal cancer COLO205 and HCT116, Chinese hamster ovary (CHO)-K1, and mouse myeloma P3X63Ag8U.1 (P3U1) cell lines were obtained from the American Type Culture Collection (ATCC, Manassas, VA, USA). Another human colorectal cancer cell line, COLO201, was obtained from the Cell Resource Center for Biomedical Research, Institute of Development, Aging, and Cancer, Tohoku University (Miyagi, Japan). CHO-K1, P3U1, and CDH-overexpressed CHO-K1 (e.g., CHO/CDH17), COLO201, COLO205, HCT116, LN229, and CDH17-overexpressed LN229 (LN229/CDH17) were cultured as described previously [23].

CDH17-KO COLO205 (BINDS-86) was generated using the CRISPR/Cas9 system. A CDH17-specific sgRNA targeting the sequence 5′- CAACTGGATATGGCCAAGAG -3′ (TrueGuide™ Synthetic sgRNA, Thermo Fisher Scientific, Inc., Waltham, MA, USA) was synthesized and cloned into the GeneArt™ CRISPR Nuclease OFP Vector (Thermo Fisher Scientific, Inc.).

### 2.2. Establishment of Cadherin–Overexpressed Stable Transfectants

Full-length CDH17 cDNA (NM_004063.4) was obtained from OriGene Technologies, Inc. (Rockville, MD, USA). The CDH17 cDNA was cloned into the pCMV6 vector. The vector was transfected into LN229 or CHO-K1 cells using the Neon transfection system (Thermo Fisher Scientific, Inc.). Stable transfectants were sorted using an anti-CDH17 mAb (clone CDH17/2618, Novus Biologicals, Centennial, CO, USA). Finally, LN229/CDH17 and CHO/CDH17 were established. Type I cadherin-overexpressed CHO-K1: CHO/CDH1, CHO/CDH2 (CHO/PA16-CDH2), CHO/CDH3, CHO/CDH4 (CHO/PA16-CDH4), and CHO/CDH15 (CHO/PA16-CDH15) were previously established [23]. Type II cadherin-overexpressed CHO-K1: CHO/CDH5 (CHO/PA16-CDH5), CHO/CDH6, CHO/CDH7 (CHO/PA16-CDH7), CHO/CDH8 (CHO/PA16-CDH8), CHO/CDH9 (CHO/PA16-CDH9), CHO/CDH10 (CHO/PA16-CDH10), CHO/CDH11 (CHO/PA16-CDH11), CHO/CDH12 (CHO/PA16-CDH12), CHO/CDH18 (CHO/PA16-CDH18), CHO/CDH19 (CHO/PA16-CDH19), CHO/CDH20 (CHO/PA16-CDH20), CHO/CDH22 (CHO/PA16-CDH22), and CHO/CDH24 (CHO/PA16-CDH24) were established previously [27]. A truncated cadherin-overexpressed CHO-K1: CHO/CDH13 (CHO/PA16-CDH13), another 7D cadherin-overexpressed CHO-K1: CHO/CDH16 (CHO/PA16-CDH16), and an atypical cadherin-overexpressed CHO-K1: CHO/CDH26 (CHO/PA16-CDH26) were previously established [27].

Each cadherin expression was confirmed using an anti-CDH1 mAb (clone 67A4, BD Biosciences, Franklin Lakes, NJ, USA), an anti-CDH3 mAb (clone MM0508-9V11, Abcam, Cambridge, UK), an anti-CDH6 mAb (clone 427909, R&D Systems Inc., Minneapolis, MN, USA), an anti-CDH13 mAb (clone 392411, R&D Systems Inc.), an anti-CDH17 mAb (clone CDH17/2618), and an anti-PA16-tag mAb (clone NZ-33) to detect other cadherins.

### 2.3. Hybridoma Production

The animal experiment was approved by the Animal Care and Use Committee of Tohoku University (Permit No. 2022MdA-001) and conducted according to relevant guidelines and regulations to minimize animal suffering and distress in the laboratory. The animal was monitored daily for health during the full duration of the experiment. A reduction in more than 25% of the total body weight was defined as a humane endpoint. A 6-week-old female BALB/cAJcl mouse (CLEA Japan, Tokyo, Japan) was immunized intraperitoneally with LN229/CDH17 (1 × 10^8^ cells). For the first immunization, 2% Alhydrogel adjuvant (InvivoGen, San Diego, CA, USA) was included. This was followed by three weekly intraperitoneal injections of LN229/CDH17 (1 × 10^8^ cells) without adjuvant. Two days before splenocyte collection, a final intraperitoneal booster injection of LN229/CDH17 (1 × 10^8^ cells) was performed. Hybridomas were produced as described previously [23]. The hybridoma supernatants were screened by flow cytometry using CHO/CDH17 and parental CHO-K1 cells. The hybridoma culture supernatant containing Ca_17_Mab-5 in serum-free Hybridoma-SFM medium (Thermo Fisher Scientific, Inc.) was purified using Ab-Catcher Extra (ProteNova, Kagawa, Japan).

### 2.4. Flow Cytometry

Cells were harvested with 1 mM EDTA and washed with blocking buffer [0.1% bovine serum albumin in phosphate-buffered saline (PBS)]. The cells were incubated with primary mAbs for 30 min at 4 °C. The cells were then stained with Alexa Fluor 488-conjugated anti-mouse IgG (1:2000; Cell Signaling Technology, Inc., Danvers, MA, USA). Flow cytometric data were acquired on an SA3800 Cell Analyzer (Sony Corp., Tokyo, Japan). Cells were gated on forward scatter (FSC) and side scatter (SSC), and fluorescence intensity was analyzed using FlowJo software (ver. 10.8.1, BD Biosciences). At least two independent experiments were conducted.

### 2.5. Calculation of the Binding Affinity by Flow Cytometry

Cells were treated with serial dilutions of Ca_17_Mab-5 or CDH17/2618. The cells were stained with Alexa Fluor 488-conjugated anti-mouse IgG (1:200 dilution). The data were collected, and the geometric mean (GeoMean) was calculated in FlowJo. Three independent measurements were performed, and the fitting binding isotherms (horizontal axis, mAb concentration; vertical axis, GeoMean) determined the apparent dissociation constant (*K*_D_) values using built-in one-site binding models of GraphPad Prism 6 (GraphPad Software, Inc., La Jolla, CA, USA).

### 2.6. Western Blotting

Western blotting was performed as described previously [23]. Ca_17_Mab-5 (1 μg/mL), CDH17/2618 (1 μg/mL), and an anti-isocitrate dehydrogenase 1 (IDH1) mAb (clone RcMab-1-mG_1_, 2 μg/mL) were used as primary mAbs. Horseradish peroxidase-conjugated anti-mouse IgG (1:1000; Agilent Technologies Inc., Santa Clara, CA, USA) was used as secondary mAb. Chemiluminescence signals were developed using Pierce™ ECL Plus (Thermo Fisher Scientific, Inc.) or ImmunoStar LD (FUJIFILM Wako Pure Chemical Corporation, Osaka, Japan). The signals were imaged with ChemiDoc Touch MP (Bio-Rad Laboratories, Inc., Berkeley, CA, USA). Two independent experiments were conducted.

### 2.7. Immunohistochemistry Using Cell Blocks and Tissue Microarrays

IHC was performed using the VENTANA BenchMark ULTRA PLUS (Roche Diagnostics, Indianapolis, IN, USA). CHO-K1, CHO/CDH17, and COLO205 were fixed with 4% paraformaldehyde, and cell blocks were prepared using iPGell (Genostaff Co., Ltd., Tokyo, Japan). Formalin-fixed, paraffin-embedded (FFPE) cell sections were stained with Ca_17_Mab-5 (0.2 or 2 μg/mL) or CvMab-62 (2 μg/mL, IgG_1_ isotype control).

Colorectal cancer microarrays (CO243b, CO352a, CO353a, CO483b, and CO484b, US Biomax Inc., Rockville, MD, USA), a gastric cancer microarray (BS01012e, US Biomax Inc.), another gastric cancer microarray (PA484, US Biomax Inc.), and a normal human organ tissue microarray (FDA999x, US Biomax Inc.) were stained with Ca_17_Mab-5 (5 μg/mL or 10 μg/mL in CO243b). Staining was performed using the ultraView Universal DAB Detection Kit (Roche Diagnostics).

## 3. Results

### 3.1. Development of Anti-CDH17 mAbs by the CBIS Method

**Figure 1:**
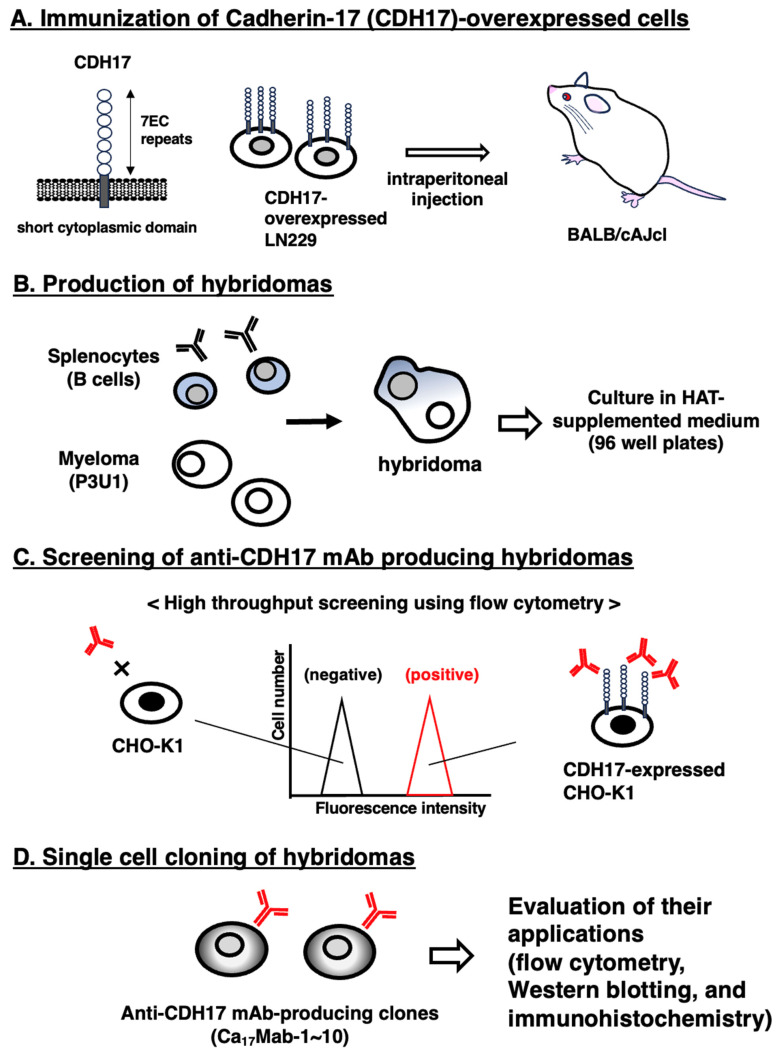
Screening of anti-CDH17 mAbs. (**A**) CDH17-overexpressed LN229 cells (LN229/CDH17) were used to immunize a BALB/cAJcl mouse. (**B**) After five immunizations, splenocytes were fused with P3U1. (**C**) Hybridoma culture supernatants were screened by flow cytometry using CHO-K1 and CHO/CDH17. (**D**) Anti-CDH17 mAb-producing hybridoma clones (Ca_17_Mabs) were established by limiting dilution.

We first prepared the immunogen LN229/CDH17 as described in Section 2.2 and Figure 1A. LN229/CDH17 (1 × 10^8^ cells) were injected intraperitoneally five times into a BALB/cAJcl mouse (Figure 1A). Splenocytes were fused with myeloma P3U1 cells to generate hybridomas, which were plated in 96-well plates (Figure 1B). Hybridoma supernatants were screened to identify those positive for CHO/CDH17 and negative for CHO-K1 (Figure 1C). Flow cytometric screening identified 129 of 474 wells (27%) that showed strong signals with CHO/CDH17 compared to CHO-K1. Limiting dilution was then performed, and a total of 10 clones producing anti-CDH17 mAbs were established. Supernatants were further evaluated for use in flow cytometry, Western blotting, and immunohistochemistry (Figure 1D). Finally, clone Ca_17_Mab-5 (IgG_1_, κ) was selected because it can be used in all applications (https://www.med-tohoku-antibody.com/topics/antibody_bank.htm, accessed on 25 June 2026).

### 3.2. Flow Cytometric Analysis of Ca_17_Mab-5 Against CHO/CDH17 and CHO-K1

**Figure 2:**
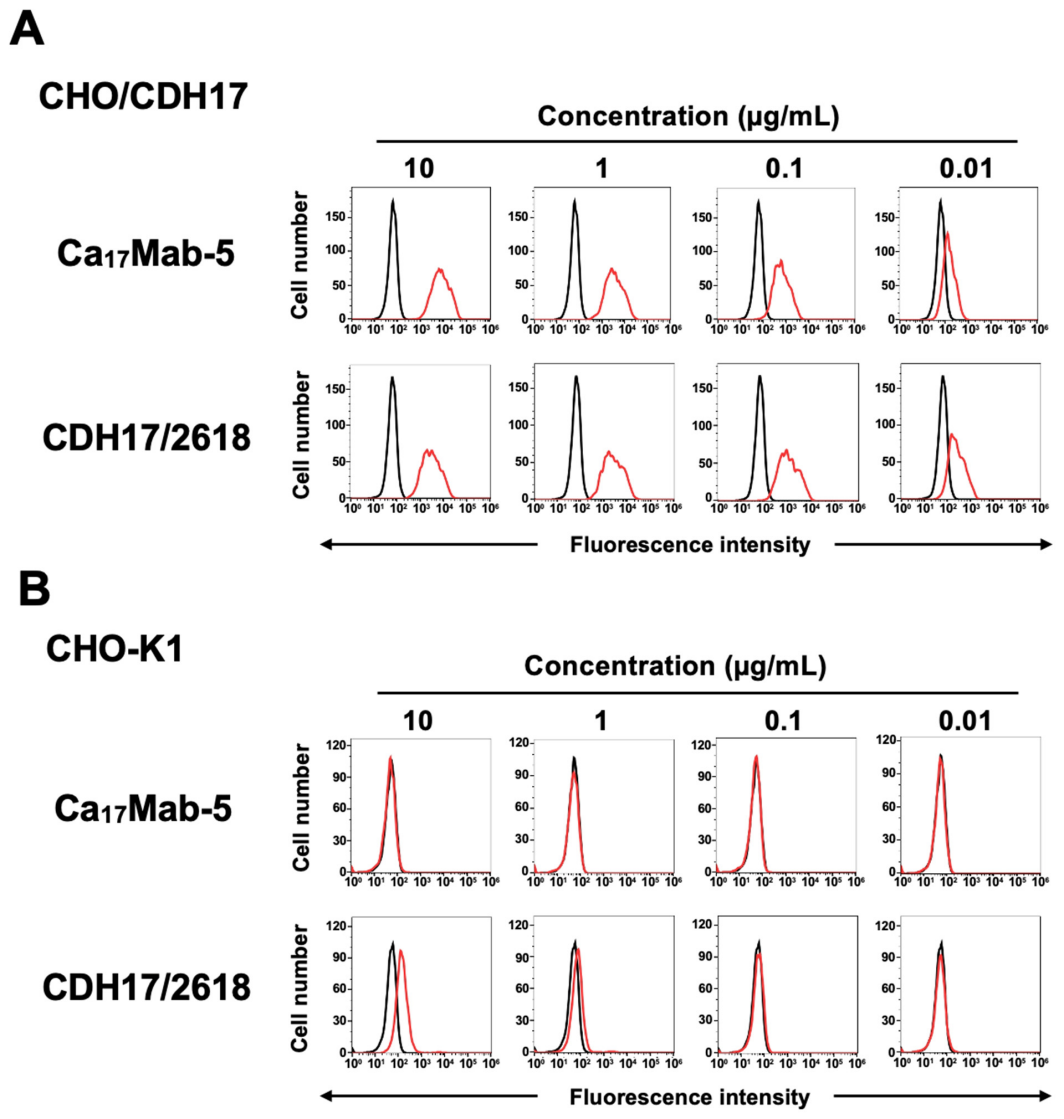
Flow cytometric analysis using Ca_17_Mab-5 and CDH17/2618. CHO/CDH17 (**A**) and CHO-K1 (**B**) were treated with Ca_17_Mab-5 or CDH17/2618 at the indicated concentrations (red line). The black line represents the negative control (blocking buffer). These cells were incubated with Alexa Fluor 488-conjugated anti-mouse IgG. Fluorescence data were collected using the SA3800 Cell Analyzer. The experiments were conducted twice.

Purified Ca_17_Mab-5 was prepared, and its reactivity was assessed by flow cytometry. As shown in Figure 2A, Ca_17_Mab-5 reacted with CHO/CDH17 in a dose-dependent manner from 10 to 0.01 μg/mL. In contrast, Ca_17_Mab-5 did not recognize parental CHO-K1 even at 10 μg/mL (Figure 2B). Although a commercially available anti-CDH17 mAb (clone CDH17/2618) reacted with CHO/CDH17 in a dose-dependent manner from 10 to 0.01 μg/mL (Figure 2A), it also non-specifically recognized CHO-K1 at 10 μg/mL (Figure 2B).

### 3.3. Determination of the Specificity of Ca_17_Mab-5 Using CDHs-Overexpressed CHO-K1

**Figure 3:**
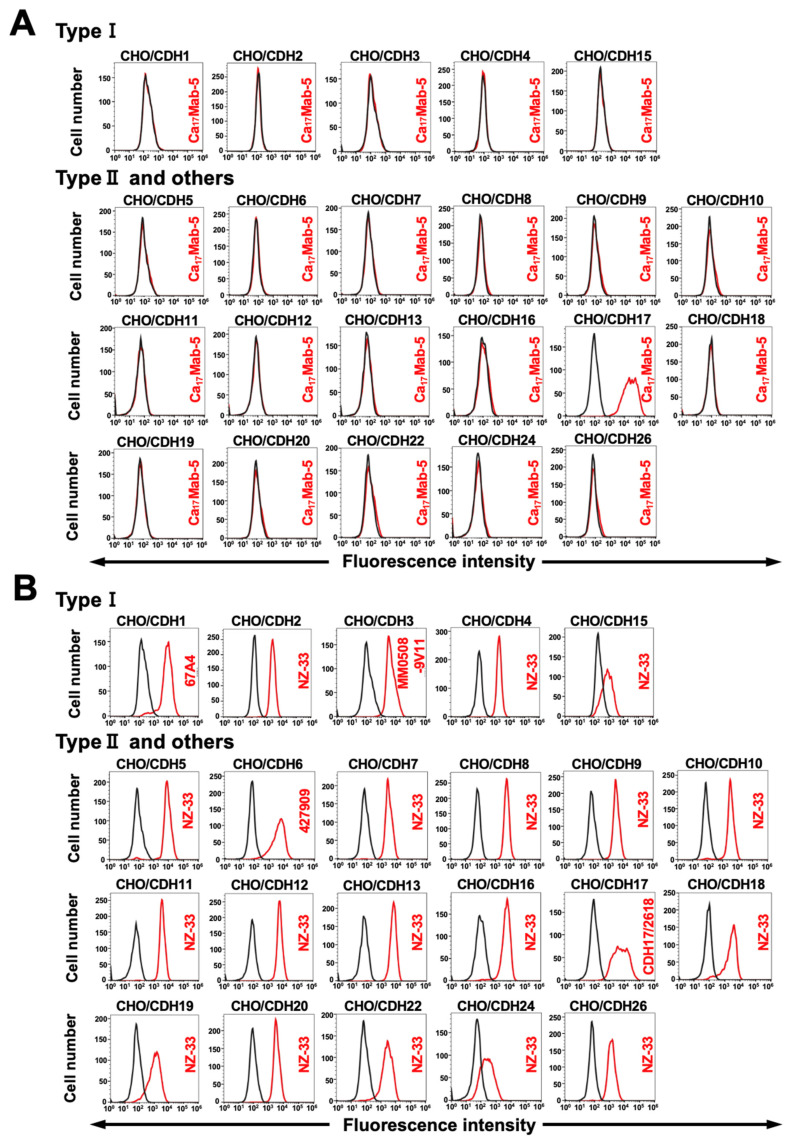
Specificity of Ca_17_Mab-5. (**A**) The type I CDHs, type II CDHs, a truncated CDH, 7D CDHs, and an atypical CDH-overexpressed CHO-K1 were treated with 10 µg/mL of Ca_17_Mab-5 (red) or with control blocking buffer (black, negative control), followed by treatment with anti-mouse IgG conjugated with Alexa Fluor 488. (**B**) Each cadherin expression was confirmed by 10 µg/mL of an anti-CDH1 mAb (clone 67A4), 10 µg/mL of an anti-CDH3 mAb (clone MM0508-9V11), 1 µg/mL of an anti-CDH6 mAb (clone 427909), 1 µg/mL of an anti-CDH17 mAb (clone CDH17/2618), and 0.1 µg/mL of an anti-PA16-tag mAb (clone NZ-33) to detect other CDHs, followed by the treatment with Alexa Fluor 488-conjugated secondary mAbs. The fluorescence data were collected using the SA3800 Cell Analyzer. The experiments were conducted twice.

We previously generated CHO-K1 cells that overexpressed type I CDHs [23,26], type II CDHs, another 7D CDH, and an atypical CDH [27]. Therefore, the specificity of Ca_17_Mab-5 to those CDHs was investigated. As shown in Figure 3A, Ca_17_Mab-5 recognized CHO/CDH17 but did not cross-react with other CDHs-overexpressed in CHO-K1. The cell surface expression of each CDH was confirmed in Figure 3B. These results indicated that Ca_17_Mab-5 is a specific mAb to CDH17 among those CDHs.

### 3.4. Flow Cytometric Analysis of Ca_17_Mab-5 and CDH17/2618 Against Endogenous CDH17-Positive and Negative Cells

**Figure 4:**
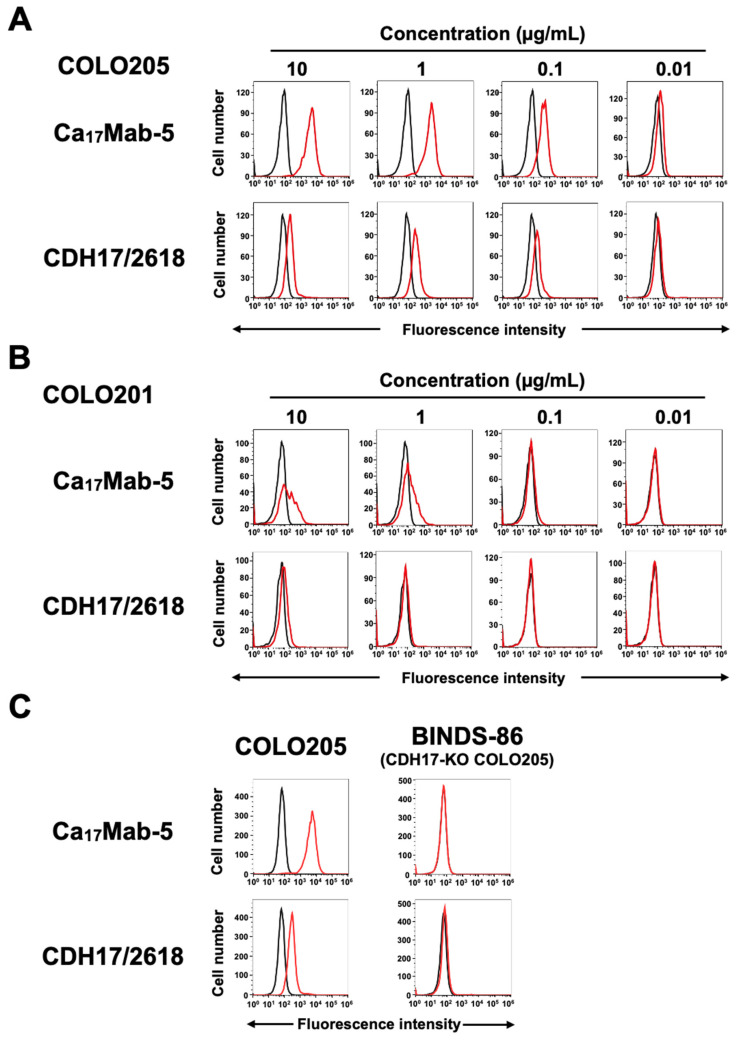
Flow cytometric analysis using Ca_17_Mab-5 and CDH17/2618 to detect endogenous CDH17. COLO205 (**A**) and COLO201 (**B**) were treated with Ca_17_Mab-5 or CDH17/2618 at the indicated concentrations (red). (**C**) COLO205 and CDH17-KO COLO205 (BINDS-86) were treated with 10 µg/mL of Ca_17_Mab-5 and CDH17/2618 (red). The black line represents the negative control (blocking buffer). These cells were incubated with Alexa Fluor 488-conjugated anti-mouse IgG. Fluorescence data were collected using the SA3800 Cell Analyzer. The experiments were conducted twice.

We then searched the expression of CDH17 in various colorectal cancer cell lines using Ca_17_Mab-5 and CDH17/2618. As shown in Figure 4, Ca_17_Mab-5 dose-dependently recognized endogenous CDH17 in COLO205. In contrast, the reactivity of CDH17/2618 was lower than that of Ca_17_Mab-5 (Figure 4A). Ca_17_Mab-5 exhibited a weak reactivity to COLO201, whereas CDH17/2618 barely reacted with COLO201 (Figure 4B). Additionally, Ca_17_Mab-5 and CDH17/2618 hardly recognized HCT116 (Supplementary Figure S1). Furthermore, we generated CDH17-KO COLO205 (BINDS-86). As shown in Figure 4C, the reactivity of Ca_17_Mab-5 completely disappeared in BINDS-86.

**Figure 5:**
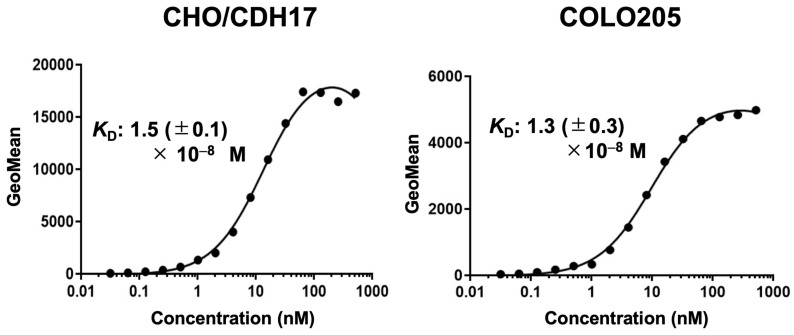
Determination of the binding affinity of Ca_17_Mab-5 by flow cytometry. CHO/CDH17 and COLO205 cells were incubated with serially diluted Ca_17_Mab-5, then reacted with Alexa Fluor 488-conjugated anti-mouse IgG. Geometric mean fluorescence values were measured using the SA3800 Cell Analyzer and FlowJo software. Average *K*_D_ values (±standard deviation) from three independent measurements were calculated using GraphPad PRISM 6. Representative graphs are shown.

The binding affinity of Ca_17_Mab-5 was measured with CHO/CDH17 and COLO205 using flow cytometry. The apparent *K*_D_ values for Ca_17_Mab-5 with CHO/CDH17 and COLO205 were 1.5 (±0.1) × 10^−8^ M and 1.3 (±0.3) × 10^−8^ M, respectively (Figure 5). Supplementary Figure S2 showed the additional information on three independent binding affinity measurements of Ca_17_Mab-5. The apparent *K*_D_ value of CDH17/2618 for COLO205 could not be determined because the sigmoid curve did not reach a plateau (Supplementary Figure S3). These results showed that Ca_17_Mab-5 has moderate binding affinity for exogenous and endogenous CDH17.

### 3.5. Detection of Exogenous and Endogenous CDH17 by Ca_17_Mab-5 and CDH17/2618 in Western Blotting

**Figure 6:**
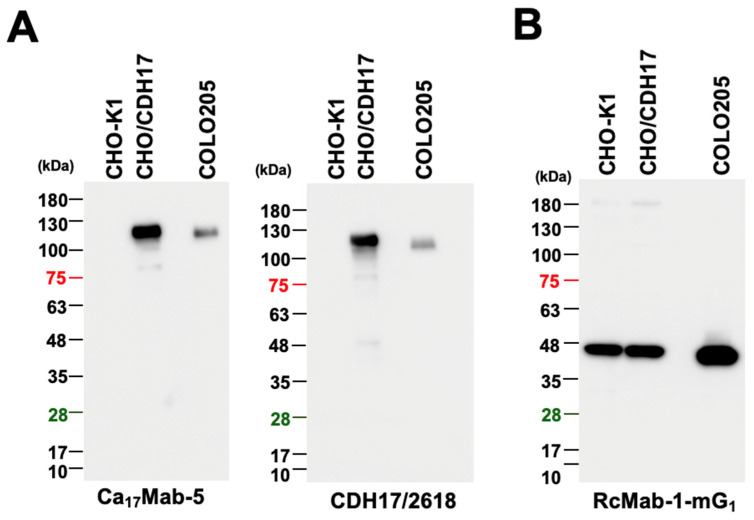
Western blotting using Ca_17_Mab-5 and CDH17/2618. Cell lysates (10 μg/lane) from CHO-K1, CHO/CDH17, and COLO205 were electrophoresed and transferred to polyvinylidene difluoride membranes. (**A**) The membranes were incubated with 1 μg/mL of Ca_17_Mab-5 or 1 μg/mL of CDH17/2618. The detection conditions were the same. (**B**) An anti-IDH1 mAb (clone RcMab-1-mG_1,_ 2 μg/mL) was used as an internal control. The membranes were further treated with anti-mouse IgG-conjugated with horseradish peroxidase. The experiments were conducted twice.

We next tested whether Ca_17_Mab-5 and CDH17/2618 can be used in Western blotting. As shown in Figure 6A, Ca_17_Mab-5 and CDH17/2618 detected a band of approximately 120 kDa in CHO/CDH17 cell lysates, whereas no band was detected in parental CHO-K1 cell lysates. Additionally, Ca_17_Mab-5 and CDH17/2618 detected endogenous CDH17 in COLO205 cell lysates at 120 kDa (Figure 6A). An anti-IDH1 mAb (clone RcMab-1-mG_1_) served as an internal control (Figure 6B). These results demonstrated that Ca_17_Mab-5 and CDH17/2618 can detect CDH17 by Western blotting.

### 3.6. Immunohistochemistry Using Ca_17_Mab-5 in Formalin-Fixed Paraffin-Embedded Cell Blocks

**Figure 7:**
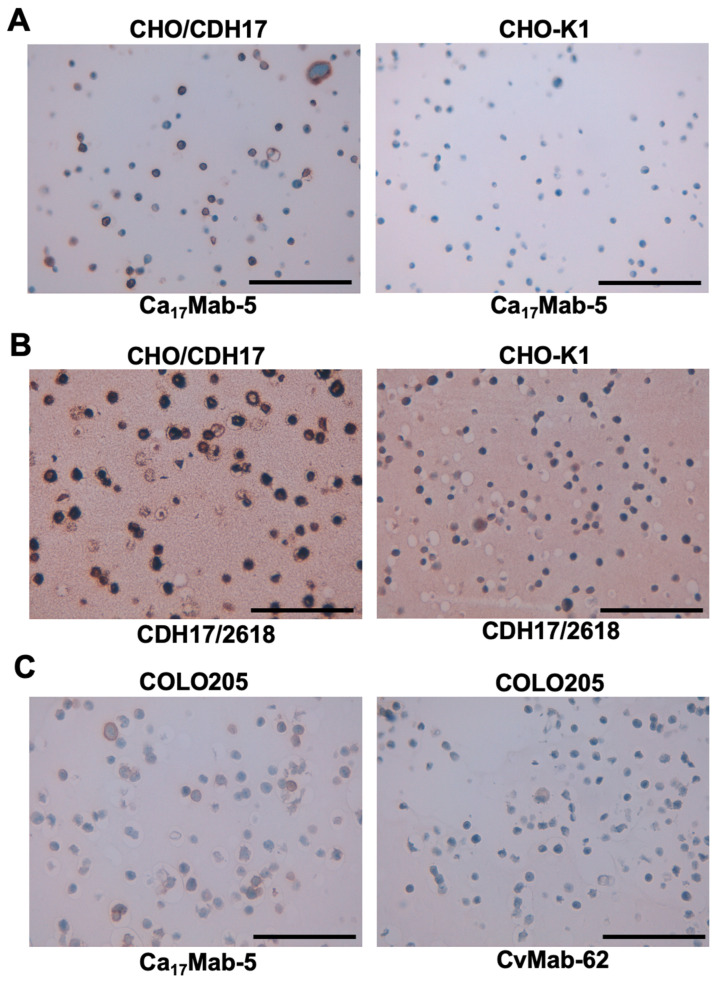
Immunohistochemistry using Ca_17_Mab-5 and CDH17/2618 in formalin-fixed paraffin-embedded cell blocks. (**A**) CHO/CDH17 and CHO-K1 sections were treated with 0.2 μg/mL of Ca_17_Mab-5. (**B**) CHO/CDH17 and CHO-K1 sections were treated with 0.2 μg/mL of CDH17/2618. (**C**) COLO205 sections were treated with 2 μg/mL of Ca_17_Mab-5 or 2 μg/mL of CvMab-62 (IgG_1_ isotype control). The staining was performed using VENTANA BenchMark ULTRA PLUS with the ultraView Universal DAB Detection Kit. Scale bar = 100 μm. The experiments were conducted twice.

We tested whether Ca_17_Mab-5 and CDH17/2618 are suitable for IHC in FFPE sections from CHO-K1 and CHO/CDH17. Ca_17_Mab-5 showed clear membranous staining in CHO/CDH17 but not in CHO-K1 (Figure 7A). In the same experimental setting, CDH17/2618 showed potent reactivity to CHO/CDH17, but weak reactivity to CHO-K1 was also detected (Figure 7B). Furthermore, Ca_17_Mab-5 also displayed membranous staining in COLO205, but the isotype control mAb (CvMab-62) did not (Figure 7C). These results indicated that Ca_17_Mab-5 can detect both exogenous and endogenous CDH17 in IHC of FFPE sections from cultured cells.

### 3.7. Immunohistochemistry Using Ca_17_Mab-5 in Normal and Tumor Microarrays

**Figure 8:**
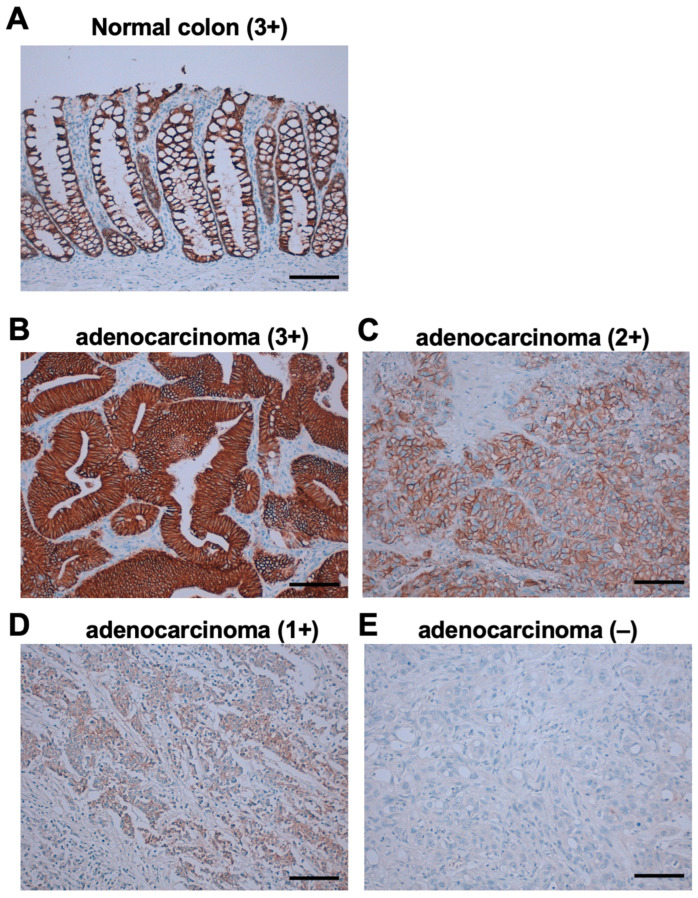
Immunohistochemistry using Ca_17_Mab-5 in colorectal cancer microarrays. The colorectal cancer microarrays were treated with 5 μg/mL of Ca_17_Mab-5. (**A**) Normal colon epithelium (strong intensity, 3+). The representative images of colorectal adenocarcinoma with strong intensity (3+, (**B**)), moderate intensity (2+, (**C**)), weak intensity (1+, (**D**)), and no staining (–, (**E**)) were shown. The staining was performed using VENTANA BenchMark ULTRA PLUS with the ultraView Universal DAB Detection Kit. Scale bar = 100 μm.

CDH17 expression is reported to be restricted to the small intestinal and colonic epithelium [9]. We investigated the CDH17 expression by Ca_17_Mab-5 using a human normal organ tissue microarray. As shown in Supplementary Figure S4, Ca_17_Mab-5 showed a potent membranous staining in small intestinal and colonic epithelia, and the weak staining was also observed in portions of normal pancreas. We next stained colorectal cancer microarrays. As shown in Figure 8A, Ca_17_Mab-5 exhibited a strong basolateral membranous staining (3+) in normal colon epithelium. In colorectal adenocarcinomas, Ca_17_Mab-5 showed strong membranous staining (3+) in well-differentiated adenocarcinomas (Figure 8B). The abundance of CDH17 detected by Ca_17_Mab-5 was reduced in poorly differentiated adenocarcinomas [Figure 8C (moderate intensity, 2+) and D (weak intensity, 1+)]. Adenocarcinomas with no CDH17 expression were also observed (Figure 8E). Table 1 summarizes the result of colorectal cancer microarray (CO483b), which includes Figure 8B–E. Supplementary Table S1 summarizes the results of other colorectal cancer tissue microarrays. Consequently, Ca_17_Mab-5 stained 133 out of 154 cases (86%, more than +1).

**Figure 9:**
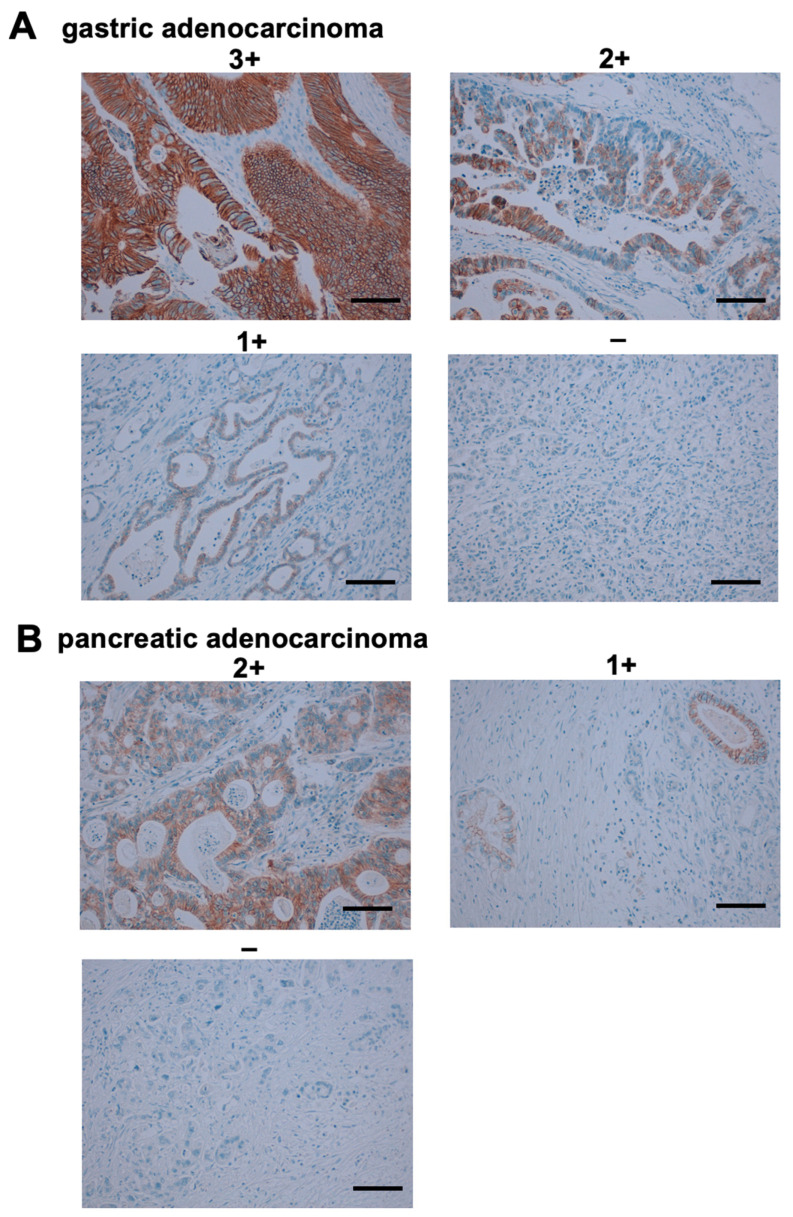
Immunohistochemistry using Ca_17_Mab-5 in gastric and pancreatic cancer microarrays. A gastric cancer microarray (BS01012e, (**A**)) and a pancreatic cancer microarray (PA484, (**B**)) were treated with 5 μg/mL of Ca_17_Mab-5. The representative images of adenocarcinomas with strong intensity (3+), moderate intensity (2+), weak intensity (1+), and no staining (−) were shown. The staining was performed using VENTANA BenchMark ULTRA PLUS with the ultraView Universal DAB Detection Kit. Scale bar = 100 μm.

We further stained gastric and pancreatic cancer microarrays using Ca_17_Mab-5. As shown in Figure 9A, Ca_17_Mab-5 also exhibited potent membranous staining in well-differentiated gastric adenocarcinomas, and moderate/weak staining was observed. In pancreatic cancer, moderate and weak membranous staining were also observed (Figure 9B). Supplementary Tables S2 and S3 summarize the results of gastric and pancreatic cancer microarrays, respectively. Consequently, Ca_17_Mab-5 stained 20 out of 72 cases (28%) in gastric cancers and 8 out of 22 cases (36%) in pancreatic cancers. These results indicated that Ca_17_Mab-5 is suitable for detecting CDH17 in IHC.

## 4. Discussion

CDH17 is expressed almost exclusively in intestinal epithelial cells of the embryonic and adult small intestine and colon [28]. Consistent with this restricted tissue distribution, CDH17 is regarded as a selective marker of colon cancer cell lines and is expressed in 35 out of 54 epithelial-like colon cancer cell lines included in the Cancer Cell Line Encyclopedia [29]. In this study, we developed novel anti-CDH17 mAbs by immunizing a mouse with CDH17-overexpressed LN229 cells (Figure 1). A clone Ca_17_Mab-5 recognized both exogenous and endogenous CDH17 in flow cytometry (Figure 2 and Figure 4) and IHC (Figure 7 and Figure 8). The information on tumor differentiation and grade was not available in the microarrays used in this study, which restricts the biological interpretation of the immunohistochemical findings. Additionally, there was no correlation between the IHC score and the T stage (*p*-value = 0.42).

Ca_17_Mab-5 was able to detect CDH17 in COLO205 but not HCT116 (Figure 4 and Figure S1), which is consistent with the gene expression profiles of colorectal cancer cell lines in the NCI-60 human cancer cell line panel [29]. Importantly, Ca_17_Mab-5 specifically recognized CDH17 without detectable cross-reactivity to other 21 cadherins, including classical type I and type II CDHs, and other types of CDHs (Figure 3). Therefore, Ca_17_Mab-5 will be useful for the specific isolation of CDH17-positive cells via fluorescence-activated cell sorting for basic research.

A commercially available anti-CDH17 mAb (clone CDH17/2618) was developed by immunization of a recombinant fragment (amino acids 242–418) of human CDH17 (https://www.novusbio.com/products/cadherin-17-antibody-cdh17-2618_nbp2-79723?srsltid=AfmBOoqdTWDqYHSRLjuVYR-gDiwQheUveNYpDjFFCxpS_btPMehtSecp#datasheet, accessed on 25 June 2026). CDH17/2618 similarly detected both exogenous and endogenous CDH17 in Western blotting (Figure 6). However, the reactivity to endogenous CDH17 in flow cytometry was low compared to that of Ca_17_Mab-5 (Figure 4A). Weak reactivities that appear to be in the background were observed at 10 µg/mL (Figure 2B and Figure 4). This may be a reason why the sigmoid curve of CDH17/2618 did not reach a plateau (Supplementary Figure S3). The strategy of CDH17/2618 selection was not described on the above website. Since Ca_17_Mab-5 was selected by a flow cytometry-based screening (Figure 1C), Ca_17_Mab-5 showed a reactivity to COLO205, which is thought to be important to target tumor cells in vivo for the development of therapeutic mAb. Furthermore, Ca_17_Mab-5 can be used for IHC of cell specimens (Figure 7) and tissue microarrays (Figure 8). Notably, IHC was conducted by an automated slide-staining system, VENTANA BenchMark ULTRA PLUS, which ensures reproducible staining conditions and accurate assessment of target expression for diagnosis.

CDH17 is aberrantly expressed not only in colorectal cancer [11,30] but also in gastric cancer [31,32], ovarian cancer [33], hepatocellular carcinoma [34], and pancreatic neuroendocrine tumors [35]. We confirmed the expression of CDH17 in gastric and pancreatic cancers by Ca_17_Mab-5 (Figure 9). CDH17 transcription is regulated by CDX2, an intestine-specific caudal-related homeobox transcription factor that plays an important role in the regulation of intestinal epithelium homeostasis [36,37]. CDX2 also mediates gastric intestinal metaplasia [38,39], a precancerous lesion defined by the replacement of gastric mucosa with intestinal-like epithelium, with a considerable risk for gastric cancer without effective therapeutic strategies [40,41,42]. There is no information that the CDH17-positive gastric cancers in Figure 9A were derived from gastric intestinal metaplasia. The pathological analysis of CDH17 expression in gastric cancer development from metaplasia is thought to be important.

Functionally, CDH17 has been shown to be a critical regulator of the stemness and chemoresistance through upregulation of the LGR5/Wnt/MYC axis and the glutamine transporter SLC38A5, respectively [17]. An anti-CDH17 RGD mAb (clone 6.6.1), an inhibitor of the α2β1 integrin-CDH17 interaction, suppressed the LGR5 expression and downstream Wnt signaling activity, suggesting that the CDH17-α2β1 integrin interaction promotes the acquisition of stemness in colorectal cancer cells [17,43]. Since the binding epitope of Ca_17_Mab-5 has not been identified, further studies are essential to determine its epitope and the biological activities of Ca_17_Mab-5. We previously published several methods to identify the binding epitope of membrane proteins using flow cytometry [44], which will aid the characterization of Ca_17_Mab-5 and other Ca_17_Mab clones in the future.

To target CDH17-positive tumors, multiple therapeutic strategies, including mAb monotherapy [43], bispecific Abs such as TRAILR2 × CDH17 [45,46] and CDH3 × CDH17 [47], ADCs [48], immunotoxins [49], radiolabeled agents [50], CAR-T [51,52], and CAR-NK cell therapies [53], have been developed in preclinical studies. Some of these modalities have been evaluated in clinical trials [54]. We previously cloned mAb cDNAs from hybridomas and generated recombinant human IgG_1_ mAbs to enhance antibody-dependent cellular cytotoxicity (ADCC), and evaluated the antitumor efficacy in human tumor xenograft models [55]. We have successfully cloned the cDNA encoding Ca_17_Mab-5, and an isotype-converted human IgG_1_-type Ca_17_Mab-5 will be generated. In a future study, we will assess the antitumor efficacy using in vitro ADCC assays and tumor xenograft models.

As shown in Figure 8A and Figure S4, and previous observation, CDH17 is predominantly localized to the basolateral membrane of normal intestinal epithelium [52]. This restricted distribution is thought to minimize ADC or CAR-T cell access to normal tissues, thereby reducing the risk of on-target off-tumor toxicities [48,52]. However, there are no preclinical models that accurately predict anti-CDH17 therapy-associated toxicities, raising concerns about on-target off-tumor toxicities in the clinical application of Ca_17_Mab-5. Adverse effects, including diarrhea and mucositis, could narrow the therapeutic window for clinical applications.

To achieve a favorable therapeutic window with reducing on-target off-tumor toxicities, we have developed cancer-specific mAbs (CasMabs) directed against several tumor-associated antigens, such as human epidermal growth factor receptor 2 (HER2) [56], and successfully identified a cancer-specific epitope of HER2. An anti-HER2 CasMab, H_2_CasMab-2, was screened based on the cancer-specific binding profile in flow cytometry [56]. H_2_CasMab-2 recognized HER2-positive breast cancer cells, while exhibiting no detectable reactivity toward various normal cells [56]. Additionally, we elucidated the structural basis of the interaction between H_2_CasMab-2 and the extracellular domain IV in the HER2 ectodomain [57]. Furthermore, a single-chain variable fragment (scFv) derived from H_2_CasMab-2 was incorporated into chimeric antigen receptor (CAR) T cells, which exhibited cancer-selective reactivity and potent antitumor activity in preclinical models [57]. H_2_CasMab-2-derived CAR-T therapy has been evaluated in a phase I clinical trial for patients with HER2-positive advanced solid tumors (NCT06241456). Therefore, these findings underscore the importance of developing CasMabs against CDH17. Moreover, defining their cancer-specific epitopes is a critical step toward the establishment of safe and effective therapeutic anti-CDH17 CasMabs and related modalities. For this purpose, we are going to increase the clones of Ca_17_Mabs and screen CasMabs against CDH17.

## Figures and Tables

**Table 1:** Immunohistochemistry of a colorectal cancer microarray (CO483b) by Ca_17_Mab-5.

No.	Age	Sex	Pathology Diagnosis	TNM	Ca_17_Mab-5
1	67	M	Adenocarcinoma	T2N0M0	3+
2	67	M	Adenocarcinoma	T3N1M0	3+
3	48	M	Adenocarcinoma	T3N0M0	3+
4	58	M	Adenocarcinoma	T3N1M0	3+
5	75	M	Adenocarcinoma with necrosis	T3N0M0	3+
6	86	M	Adenocarcinoma	T4N1M0	3+
7	55	M	Adenocarcinoma	T2N0M0	3+
8	38	M	Adenocarcinoma	T4N1M0	3+
9	52	M	Adenocarcinoma	T3N1M0	3+
10	46	M	Mucinous adenocarcinoma	T3N2M0	3+
11	61	M	Mucinous adenocarcinoma (Figure 8B)	T3N0M0	3+
12	55	M	Adenocarcinoma	T3N2M0	2+
13	46	F	Adenocarcinoma	T3N1M0	3+
14	44	M	Adenocarcinoma	T4N1M0	2+
15	31	M	Adenocarcinoma	T4N1M0	2+
16	74	F	Adenocarcinoma	T4N0M0	3+
17	61	M	Adenocarcinoma (Figure 8E)	T3N0M0	−
18	45	M	Adenocarcinoma	T4N1M0	1+
19	58	M	Mucinous adenocarcinoma	T3N0M0	2+
20	78	M	Adenocarcinoma with necrosis	T3N1M1	3+
21	69	M	Adenocarcinoma	T4N2M0	2+
22	64	F	Adenocarcinoma (Figure 8C)	T4N1M0	2+
23	82	M	Mucinous adenocarcinoma	T4N0M0	2+
24	34	M	Adenocarcinoma (Figure 8D)	T3N0M0	1+
25	50	F	Adenocarcinoma	T4N0M0	2+
26	34	F	Adenocarcinoma	T3N0M0	1+
27	52	F	Adenocarcinoma	T3N2M0	2+
28	53	F	Adenocarcinoma	T2N0M0	2+
29	58	F	Adenocarcinoma	T3N0M0	−
30	59	F	Adenocarcinoma	T3N1M0	1+
31	67	M	Adenocarcinoma	T3N2M0	1+
32	31	M	Adenocarcinoma	T4N0M0	−
33	54	F	Adenocarcinoma	T4N1M0	−
34	62	M	Adenocarcinoma	T2N0M0	1+
35	80	M	Adenocarcinoma	T4N2M0	2+
36	67	F	Adenocarcinoma	T3N0M0	1+
37	52	F	Adenocarcinoma	T3N1M0	1+
38	73	M	Adenocarcinoma	T3N0M0	−
39	75	M	Adenocarcinoma	T4N0M0	−
40	57	F	Adenocarcinoma	T2N0M0	2+
41	30	M	Sigmoid colon tissue	normal	2+
42	22	M	Sigmoid colon tissue	normal	3+
43	30	M	Colon tissue	normal	3+
44	35	M	Colon tissue	normal	3+
45	45	M	Colon tissue	normal	3+
46	32	M	Ascending colon tissue	normal	3+
47	32	M	Colon tissue	normal	3+

−, no stain; 1+, weak intensity; 2+, moderate intensity; 3+, strong intensity.

## Data Availability

The data presented in this study are available in the article.

## Supplementary Materials

The following supporting information can be downloaded at https://www.mdpi.com/article/10.3390/antib15040059/s1. Figure S1: Flow cytometric analysis using Ca_17_Mab-5 and CDH17/2618 to detect endogenous CDH17 in HCT116. Figure S2: Additional information on three independent binding affinity measurements of Ca_17_Mab-5 by flow cytometry. Figure S3: Determination of binding affinity of CDH17/2618 by flow cytometry. Figure S4: Immunohistochemistry using Ca_17_Mab-5 (5 μg/mL) in a human normal organ tissue microarray. Table S1: Immunohistochemistry of colorectal cancer microarrays by Ca_17_Mab-5. Table S2: Immunohistochemistry of a gastric cancer microarray (BS01012e) by Ca_17_Mab-5. Table S3: Immunohistochemistry of a pancreatic cancer microarray (PA484) by Ca_17_Mab-5.
