# Supplementary material for: A Novel Anti-Cadherin-17 Monoclonal Antibody, Ca17Mab-5, for Multiple Applications
Source: Antibodies (Basel). 2026 Jul 10;15(4):59. doi: 10.3390/antib15040059 (PMC13398008; doi:10.3390/antib15040059)
Supplement: Supplementary file 1 [file antibodies-15-00059-s001.zip › final supple Fig. S1-S4.pdf]

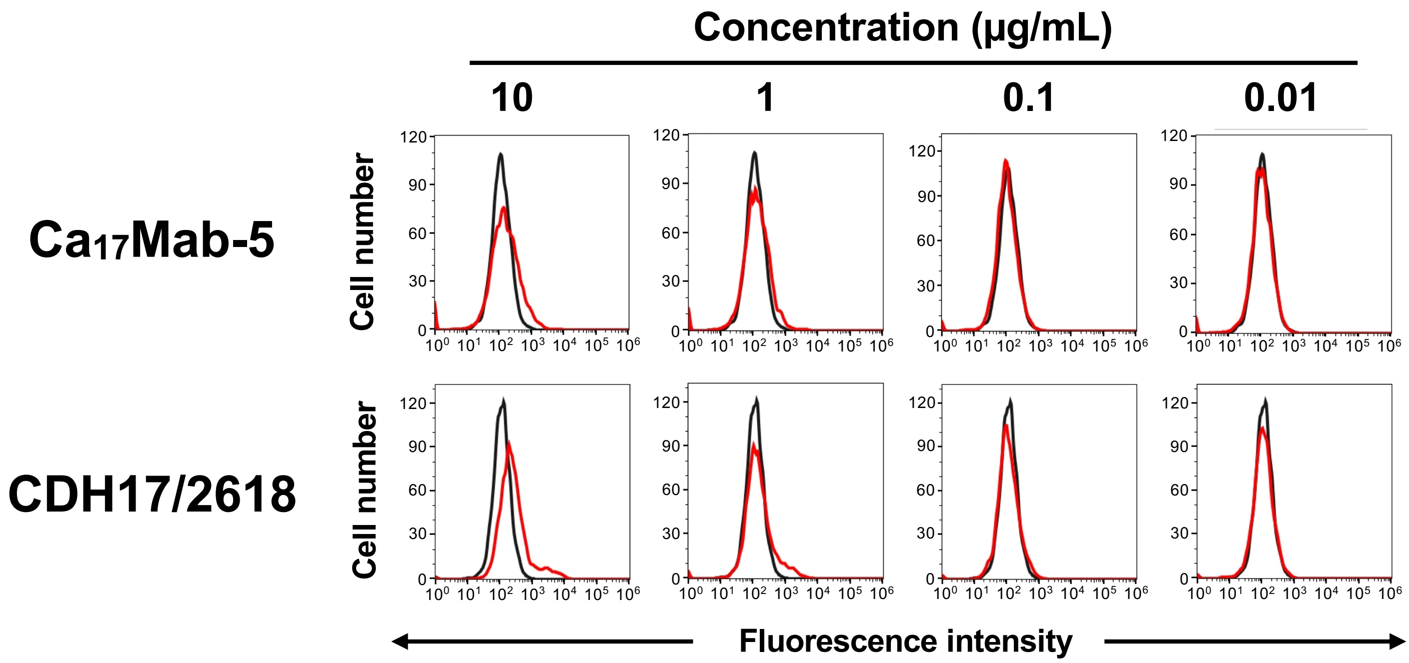

Supplementary Figure S1. Flow cytometric analysis using Ca_{17}Mab-5 and CDH17/2618 to detect endogenous CDH17 in HCT116. HCT116 was treated with Ca_{17}Mab-5 and CDH17/2618 at the indicated concentrations (red) or blocking buffer (black, negative control). These cells were incubated with Alexa Fluor 488-conjugated anti-mouse IgG. Fluorescence data were collected using the SA3800 Cell Analyzer.

CHO/CDH17

One site -- Total	
Best-fit values	
Bmax	16611
Kd	15.91
NS	-0.6849
Background	-134.5
Std. Error	
Bmax	323.6
Kd	1.172
NS	0.394
Background	142.6
95% Confidence Intervals	
Bmax	15905 to 17316
Kd	13.36 to 18.47
NS	-1.543 to 0.1736
Background	-445.3 to 176.2
Goodness of Fit	
Degrees of Freedom	12
R square	0.998
Absolute Sum of Squares	1297000
Sy.x	328.8

One site -- Total	
Best-fit values	
Bmax	15039
Kd	14.83
NS	-0.7212
Background	-155.5
Std. Error	
Bmax	365.1
Kd	1.38
NS	0.4487
Background	166.1
95% Confidence Intervals	
Bmax	14243 to 15834
Kd	11.83 to 17.84
NS	-1.699 to 0.2564
Background	-517.4 to 206.4
Goodness of Fit	
Degrees of Freedom	12
R square	0.9968
Absolute Sum of Squares	1730000
Sy.x	379.6

One site -- Total	
Best-fit values	
Bmax	20444
Kd	13.63
NS	-5.913
Background	-129.6
Std. Error	
Bmax	803.2
Kd	1.716
NS	2.136
Background	257.2
95% Confidence Intervals	
Bmax	18676 to 22211
Kd	9.854 to 17.41
NS	-10.62 to -1.211
Background	-695.7 to 436.6
Goodness of Fit	
Degrees of Freedom	11
R square	0.9950
Absolute Sum of Squares	3.844e+006
Sy.x	591.1

COLO205

One site -- Total	
Best-fit values	
Bmax	6259
Kd	14.14
NS	0.4009
Background	53.15
Std. Error	
Bmax	252.9
Kd	1.812
NS	0.6672
Background	79.12
95% Confidence Intervals	
Bmax	5703 to 6816
Kd	10.16 to 18.13
NS	-1.068 to 1.869
Background	-121.0 to 227.3
Goodness of Fit	
Degrees of Freedom	11
R square	0.9958
Absolute Sum of Squares	366775
Sy.x	182.6

One site -- Total	
Best-fit values	
Bmax	4239
Kd	15.10
NS	-1.072
Background	47.37
Std. Error	
Bmax	266.3
Kd	2.949
NS	0.6921
Background	79.83
95% Confidence Intervals	
Bmax	3653 to 4825
Kd	8.613 to 21.59
NS	-2.596 to 0.4510
Background	-128.3 to 223.1
Goodness of Fit	
Degrees of Freedom	11
R square	0.9886
Absolute Sum of Squares	379178
Sy.x	185.7

One site -- Total	
Best-fit values	
Bmax	5336
Kd	9.720
NS	-0.5901
Background	-22.20
Std. Error	
Bmax	118.9
Kd	0.7609
NS	0.3383
Background	46.56
95% Confidence Intervals	
Bmax	5075 to 5598
Kd	8.045 to 11.39
NS	-1.335 to 0.1546
Background	-124.7 to 80.27
Goodness of Fit	
Degrees of Freedom	11
R square	0.9981
Absolute Sum of Squares	115737
Sy.x	102.6

Supplementary Figure S2. Additional information on three independent binding affinity measurements of Ca_{17}Mab-5 by flow cytometry.

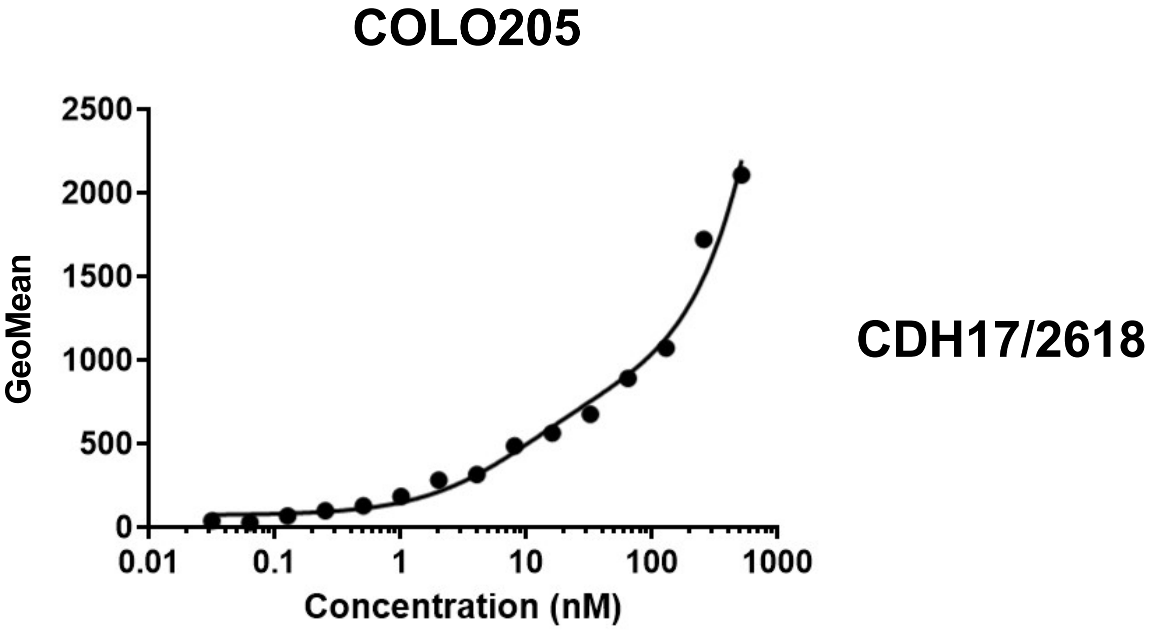

Supplementary Figure S3. Determination of binding affinity of CDH17/2618 by flow cytometry. COLO205 were suspended in serially diluted Ca_{17}Mab-5. Then, cells were reacted with Alexa Fluor 488-conjugated anti-mouse IgG. The geometric mean fluorescence values were obtained using the SA3800 Cell Analyzer and FlowJo software. The K_D value was not determined because the sigmoid curve did not reach a plateau.

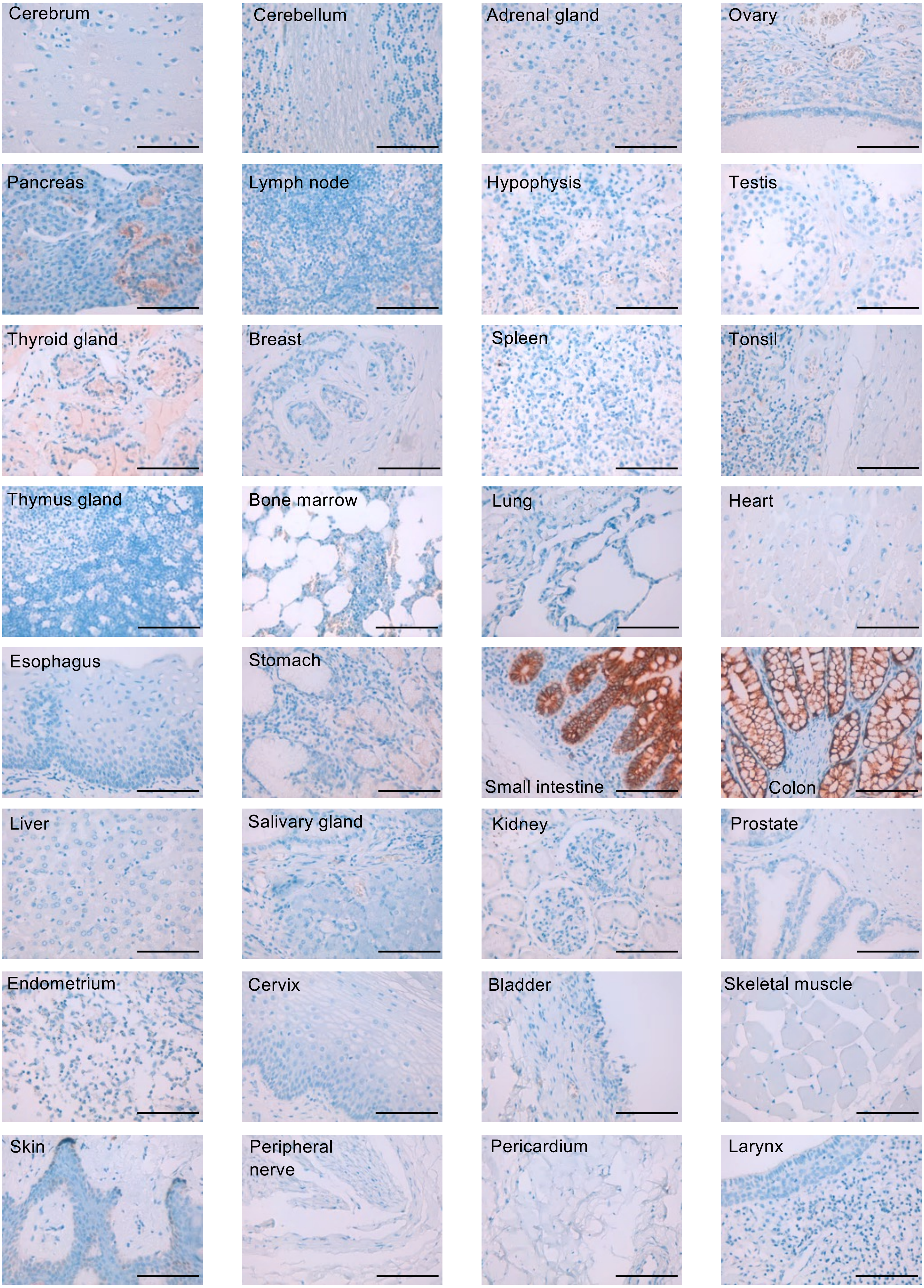

Supplementary Figure S4. Immunohistochemistry using Ca_{17}Mab-5 (5 μg/mL) in a human normal organ tissue microarray. The staining was performed using VENTANA BenchMark ULTRA PLUS with the ultraView Universal DAB Detection Kit. Scale bar = 100 μm.
