# Supplementary material for: A Novel Anti-Cadherin-17 Monoclonal Antibody, Ca17Mab-5, for Multiple Applications
Source: Antibodies (Basel). 2026 Jul 10;15(4):59. doi: 10.3390/antib15040059 (PMC13398008; doi:10.3390/antib15040059)
Supplement: Supplementary file 1 [file antibodies-15-00059-s001.zip › supple Table S1 .pdf]

Supplementary Table S1. Immunohistochemistry of colorectal cancer microarrays by Ca_{17}Mab-5.

CO352a Moderately differentiated adenocarcinoma of colon tissue array

No.	Age	Sex	Pathology diagnosis	TNM	Ca_{17}Mab-5
1	51	M	Adenocarcinoma	T2N0M0	2+
2	48	M	Adenocarcinoma	T3N0M0	2+
3	68	M	Adenocarcinoma with necrosis	T3N0M0	3+
4	60	M	Adenocarcinoma	T3N0M0	3+
5	54	M	Adenocarcinoma	T3N0M0	3+
6	53	F	Adenocarcinoma	T3N0M0	2+
7	76	M	Adenocarcinoma	T3N0M0	3+
8	46	M	Adenocarcinoma	T3N0M0	2+
9	58	M	Adenocarcinoma	T3N0M0	−
10	47	M	Adenocarcinoma	T3N0M0	1+
11	65	F	Adenocarcinoma	T3N0M0	2+
12	70	M	Adenocarcinoma	T3N0M0	3+
13	65	F	Adenocarcinoma	T3N0M0	3+
14	58	M	Adenocarcinoma	T3N0M0	3+
15	50	M	Adenocarcinoma	T3N0M0	3+
16	65	F	Adenocarcinoma	T3N0M0	3+
17	43	M	Adenocarcinoma	T3N0M0	2+
18	73	M	Adenocarcinoma	T4N0M0	3+
19	68	M	Adenocarcinoma	T4N0M0	3+
20	66	F	Adenocarcinoma	T4N0M0	3+
21	60	M	Adenocarcinoma	T4N0M0	2+
22	50	F	Adenocarcinoma	T4N0M0	3+
23	46	M	Adenocarcinoma	T3N1M0	3+
24	64	M	Adenocarcinoma	T3N1M0	3+
25	56	M	Adenocarcinoma	T3N1M0	2+
26	34	M	Adenocarcinoma	T3N1M0	3+
27	59	F	Adenocarcinoma	T3N1M0	2+
28	58	M	Adenocarcinoma	T3N1M0	3+
29	66	M	Adenocarcinoma	T3N1M0	2+
30	52	F	Adenocarcinoma	T3N2M0	3+
31	60	M	Adenocarcinoma	T3N2M0	3+
32	52	M	Adenocarcinoma	T3N2M0	3+
33	47	M	Adenocarcinoma	T4N1M0	3+
34	74	M	Adenocarcinoma	T4N2M0	3+

−, No stain; 1+, Weak intensity; 2+, Moderate intensity; 3+, Strong intensity.

Supplementary Table S1 (continued)

CO353a Colon adenocarcinoma tissue array

No.	Age	Sex	Pathology diagnosis	TNM	Ca_{17}Mab-5
1	71	M	Adenocarcinoma	T2N0M0	–
2	48	M	Adenocarcinoma	T2N0M0	2+
3	59	M	Adenocarcinoma	T3N0M0	1+
4	52	M	Adenocarcinoma	T3N0M0	–
5	65	M	Adenocarcinoma	T3N0M0	1+
6	56	F	Adenocarcinoma	T3N0M0	–
7	67	F	Adenocarcinoma	T3N0M0	–
8	47	M	Adenocarcinoma	T3N0M0	1+
9	61	M	Adenocarcinoma	T3N0M0	–
10	70	M	Adenocarcinoma	T3N0M0	3+
11	36	F	Mucinous adenocarcinoma	T3N0M0	2+
12	60	F	Adenocarcinoma	T3N0M0	–
13	77	M	Adenocarcinoma	T4N0M0	3+
14	77	M	Adenocarcinoma	T3N1M0	3+
15	57	M	Mucinous adenocarcinoma	T3N1M0	3+
16	70	M	Adenocarcinoma	T3N1M0	3+
17	44	F	Adenocarcinoma	T3N1M0	1+
18	63	F	Adenocarcinoma	T3N1M0	3+
19	69	M	Adenocarcinoma	T3N1M0	1+
20	63	M	Adenocarcinoma	T3N1M0	2+
21	41	F	Adenocarcinoma	T3N1M0	2+
22	60	F	Adenocarcinoma	T3N1M0	3+
23	34	M	Adenocarcinoma	T3N1M0	3+
24	62	M	Adenocarcinoma	T3N1M0	3+
25	62	M	Adenocarcinoma	T3N1M0	–
26	67	M	Mucinous adenocarcinoma	T3N1M0	2+
27	80	F	Adenocarcinoma	T3N1M0	–
28	78	M	Adenocarcinoma	T3N1M0	2+
29	59	M	Adenocarcinoma	T3N1M0	2+
30	38	M	Adenocarcinoma	T4N1M0	2+
31	50	M	Adenocarcinoma	T3N2M0	3+
32	56	M	Adenocarcinoma	T3N2bM0	2+
33	55	M	Adenocarcinoma	T3N2M0	2+
34	35	M	Adenocarcinoma	T4N1M0	–
35	40	M	Adenocarcinoma	T4N2M0	3+

–, No stain; 1+, Weak intensity; 2+, Moderate intensity; 3+, Strong intensity.

Supplementary Table S1 (continued)

CO484b Colon cancer tissue array with adjacent normal tissues

No.	Age	Sex	Pathology diagnosis	TNM	Ca_{17}Mab-5
1	38	M	Adenocarcinoma	T3N0M0	1+
2	49	M	Adenocarcinoma	T2N0M0	3+
3	62	M	Adenocarcinoma	T4N0M0	1+
4	58	M	Smooth muscle tissue	T4N1M0	3+
5	58	M	Adenocarcinoma	T4N1M1	2+
6	61	M	Adenocarcinoma	T3N0M0	2+
7	51	F	Adenocarcinoma	T4N1M1	3+
8	55	F	Adenocarcinoma	T3N0M0	3+
9	56	M	Adenocarcinoma	T3N0M0	3+
10	45	M	Adenocarcinoma	T3N0M0	2+
11	68	F	Adenocarcinoma	T3N0M0	2+
12	48	F	Adenocarcinoma	T3N1M0	3+
13	70	M	Adenocarcinoma	T4N1M0	−
14	50	M	Adenocarcinoma	T4N0M0	3+
15	52	M	Adenocarcinoma	T4N0M0	2+
16	34	M	Adenocarcinoma with necrosis	T3N0M0	3+
17	53	M	Adenocarcinoma	T3N2M1	2+
18	42	F	Adenocarcinoma	T4N2M0	3+
19	50	M	Mucinous adenocarcinoma	T3N0M0	3+
20	48	M	Adenocarcinoma	T4N2M0	1+
21	39	M	Adenocarcinoma	T3N0M0	2+
22	38	M	Adenocarcinoma	T4N0M0	1+
23	51	M	Adenocarcinoma	T3N2M0	1+
24	38	F	Adenocarcinoma	T4N1M0	2+
25	56	M	Mucinous adenocarcinoma	T3N2M0	2+
26	47	M	Mucinous adenocarcinoma	T3N0M0	3+
27	74	M	Mucinous adenocarcinoma	T4N0M0	3+
28	56	M	Mucinous adenocarcinoma	T3N1M0	3+
29	43	M	Mucinous adenocarcinoma	T3N0M0	3+
30	56	F	Mucinous adenocarcinoma	T3N1M0	1+
31	22	M	Mucinous adenocarcinoma	T3N0M0	3+
32	56	M	Mucinous adenocarcinoma	T3N1M0	−
33	34	M	Mucinous adenocarcinoma	T3N1M0	2+
34	31	M	Signet-ring cell carcinoma	T3N0M0	2+
35	35	M	Signet-ring cell carcinoma	T4N0M0	−
36	72	M	Signet-ring cell carcinoma	T3N0M0	−
37	16	M	Signet-ring cell carcinoma	T3N0M0	3+
38	46	M	Signet-ring cell carcinoma	T4N1M0	1+

39	75	F	Signet-ring cell carcinoma	T3N0M0	1+
40	28	M	Non-neoplastic colon tissue	normal	3+
41	30	M	Non-neoplastic colon tissue	normal	3+
42	30	M	Small amount of colon tissue	normal	3+
43	45	M	Non-neoplastic colon tissue	normal	3+
44	30	M	Non-neoplastic colon tissue	normal	3+
45	35	M	Non-neoplastic colon tissue	normal	3+

−, No stain; 1+, Weak intensity; 2+, Moderate intensity; 3+, Strong intensity.

CO243b Colon adenocarcinoma tissue array with matched normal adjacent tissue

No.	Age	Sex	Pathology diagnosis	TNM	Ca_{17}Mab-5
1	70	M	Adenocarcinoma	T3N0M0	3+
2	50	F	Adenocarcinoma	T3N1M0	3+
3	71	M	Adenocarcinoma	T3N0M0	3+
4	51	M	Adenocarcinoma	T4N2M0	3+
5	66	M	Adenocarcinoma	T3N0M0	1+
6	47	M	Adenocarcinoma	T4N0M0	3+
7	70	M	Adjacent tissue of No.1	normal	3+
8	50	F	Adjacent tissue of No.3	normal	3+
9	71	M	Adjacent tissue of No.5	normal	3+
10	23	M	Colon tissue	normal	3+
11	45	M	Colon tissue	normal	3+
12	21	F	Colon tissue	normal	3+

−, No stain; 1+, Weak intensity; 2+, Moderate intensity; 3+, Strong intensity.
