# Supplementary material for: A Novel Anti-Cadherin-17 Monoclonal Antibody, Ca17Mab-5, for Multiple Applications
Source: Antibodies (Basel). 2026 Jul 10;15(4):59. doi: 10.3390/antib15040059 (PMC13398008; doi:10.3390/antib15040059)
Supplement: Supplementary file 1 [file antibodies-15-00059-s001.zip › supple Table S2 .pdf]

Supplementary Table S2. Immunohistochemistry of a gastric cancer microarray (BS01012e) by Ca_{17}Mab-5.

No.	Age	Sex	Pathology diagnosis	TNM	Ca_{17}Mab-5
1	49	F	Adenocarcinoma	T3N1M0	1+
2	68	M	Adenocarcinoma	T3N0M0	−
3	55	M	Adenocarcinoma	T4N2M0	−
4	75	M	Adenocarcinoma	T3N1M0	−
5	60	F	Adenocarcinoma	T2N1M0	−
6	48	F	Adenocarcinoma	T3N2M0	3+
7	66	M	Adenocarcinoma	T3N0M0	−
8	55	F	Adenocarcinoma	T2N0M0	3+
9	47	F	Adenocarcinoma	T3N1M0	−
10	61	M	Adenocarcinoma	T3N1M0	−
11	70	M	Adenocarcinoma	T3N1M0	−
12	60	M	Adenocarcinoma	T2N1M0	−
13	54	M	Adenocarcinoma	T2N1M0	−
14	72	M	Adenocarcinoma	T2N1M0	−
15	71	M	Adenocarcinoma	T2N1M0	−
16	63	M	Adenocarcinoma	T2N1M0	1+
17	66	M	Adenocarcinoma	T1N2M0	1+
18	53	M	Adenocarcinoma	T3N1N0	1+
19	68	F	Adenocarcinoma	T2N1M0	−
20	64	F	Adenocarcinoma	T2N1M0	1+
21	50	F	Adenocarcinoma	T3N0M0	−
22	72	M	Adenocarcinoma	T2N0M0	−
23	50	M	Mucinous adenocarcinoma	T3N0M0	1+
24	69	M	Adenocarcinoma	T2N0M0	2+
25	59	M	Adenocarcinoma	T2N0M0	−
26	35	M	Adenocarcinoma	T3N0M0	2+
27	43	M	Adenocarcinoma	T3N1M0	−
28	60	M	Mucinous adenocarcinoma	T3N1M0	−
29	70	F	Adenocarcinoma	T2N0M0	−
30	64	M	Adenocarcinoma	T2N1M0	1+
31	45	M	Adenocarcinoma	T3N1M0	−
32	35	F	Adenocarcinoma	T3N2M0	1+
33	57	M	Adenocarcinoma	T3N1N0	−
34	72	M	Adenocarcinoma	T3N1N0	−
35	68	M	Adenocarcinoma	T3N1N0	1+
36	72	F	Adenocarcinoma	T3N0M0	−
37	62	M	Adenocarcinoma	T2N2M0	−
38	68	F	Adenocarcinoma	T2N1M0	−

39	43	M	Adenocarcinoma	T2N0M0	2+
40	55	M	Adenocarcinoma	T3N2M0	−
41	61	M	Adenocarcinoma	T3N0M0	−
42	49	F	Adenocarcinoma	T3N1M0	−
43	49	M	Adenocarcinoma	T2N1M0	1+
44	57	M	Adenocarcinoma	T3N1M0	1+
45	63	M	Adenocarcinoma	T3N2M0	−
46	59	M	Adenocarcinoma	T3N1M0	−
47	60	M	Adenocarcinoma	T2N1M0	−
48	46	F	Adenocarcinoma	T3N1M0	−
49	50	M	Adenocarcinoma	T2N1M0	−
50	63	M	Adenocarcinoma	T2N1M0	−
51	37	F	Adenocarcinoma	T2N2M0	−
52	45	M	Adenocarcinoma	T2N1M0	−
53	34	M	Adenocarcinoma	T2N1M0	−
54	54	M	Adenocarcinoma	T3N1M0	−
55	64	M	Adenocarcinoma	T2N1M0	3+
56	40	M	Adenocarcinoma	T3N2M0	−
57	68	M	Mucinous adenocarcinoma	T2N1M0	−
58	33	M	Mucinous adenocarcinoma	T3N1M0	−
59	70	M	Adenocarcinoma	T2N0M0	−
60	46	M	Mucinous adenocarcinoma	T3N0M0	−
61	55	M	Adenocarcinoma	T3N1M0	−
62	58	M	Adenocarcinoma	T2N1M0	3+
63	24	F	Adenocarcinoma	T3N1M0	−
64	35	M	Adenocarcinoma	T3N1M0	−
65	47	F	Adenocarcinoma	T3N1M0	−
66	64	M	Adenocarcinoma	T2N1M0	−
67	56	M	Adenocarcinoma	T3N2M0	2+
68	75	M	Adenocarcinoma	T2N0M0	−
69	48	M	Adenocarcinoma	T2N0M0	−
70	48	M	Adenocarcinoma	T2N0M0	1+
71	74	M	Adenocarcinoma	T2N0M0	−
72	45	F	Adenocarcinoma	T4N0M1	−

−, No stain; 1+, Weak intensity; 2+, Moderate intensity; 3+, Strong intensity.
