# Supplementary material for: A Novel Anti-Cadherin-17 Monoclonal Antibody, Ca17Mab-5, for Multiple Applications
Source: Antibodies (Basel). 2026 Jul 10;15(4):59. doi: 10.3390/antib15040059 (PMC13398008; doi:10.3390/antib15040059)
Supplement: Supplementary file 1 [file antibodies-15-00059-s001.zip › supple Table S3.pdf]

Supplementary Table S3. Immunohistochemistry of a pancreatic cancer microarray (PA484) by Ca_{17}Mab-5.

No.	Age	Sex	Pathology diagnosis	TNM	Ca_{17}Mab-5
1	35	F	Islet cell tumor	-	−
2	60	M	Adenocarcinoma	T3N0M0	1+
3	68	F	Adenocarcinoma	T2N0M0	−
4	54	F	Adenocarcinoma	T3N0M0	1+
5	42	F	Adenocarcinoma	T3N0M0	−
6	65	M	Adenocarcinoma	T3N0M0	−
7	75	F	Adenocarcinoma	T3N0M1	1+
8	57	M	Adenocarcinoma	T3N0M0	1+
9	44	M	Adenocarcinoma	T3N0M0	2+
10	47	M	Adenocarcinoma	T3N0M0	1+
11	41	M	Adenocarcinoma	T4N1M0	2+
12	64	F	Adenocarcinoma	T3N0M0	−
13	58	F	Adenocarcinoma	T3N0M0	−
14	47	F	Adenocarcinoma	T3N1M0	−
15	78	M	Adenocarcinoma	T2N0M0	−
16	49	M	Adenocarcinoma	T3N0M0	1+
17	53	F	Adenocarcinoma	T3N0M0	−
18	60	M	Adenocarcinoma	T2N0M0	−
19	57	F	Adenocarcinoma	T2N0M0	−
20	61	M	Mucinous adenocarcinoma	T3N0M1	−
21	69	M	Undifferentiated carcinoma	T2N0M0	−

−, No stain; 1+, Weak intensity; 2+, Moderate intensity; 3+, Strong intensity.
